# Reply: Retinopathy, histidine-rich protein-2 and perfusion pressure in cerebral malaria

**DOI:** 10.1093/brain/awu146

**Published:** 2014-06-11

**Authors:** Ian J. C. MacCormick, Nicholas A. V. Beare, Terrie E. Taylor, Valentina Barrera, Valerie A. White, Paul Hiscott, Malcolm E. Molyneux, Baljean Dhillon, Simon P. Harding

**Affiliations:** 1 Malawi-Liverpool-Wellcome Trust Clinical Research Programme, Blantyre, Malawi; 2 Department of Eye and Vision Science, University of Liverpool, UK; 3 St. Paul’s Eye Unit, Royal Liverpool University Hospital, Liverpool, UK; 4 College of Medicine, University of Malawi, Blantyre, Malawi; 5 Blantyre Malaria Project, Blantyre, Malawi; 6 Department of Osteopathic Medical Specialities, Michigan State University, Michigan, USA; 7 Department of Pathology and Laboratory Medicine, Vancouver General Hospital, British Columbia, Canada; 8 Liverpool School of Tropical Medicine, Liverpool, UK; 9 Department of Ophthalmology, University of Edinburgh, Edinburgh, UK; 10 Princess Alexandra Eye Pavilion, Edinburgh, UK

Sir, We thank Kariuki and Newton for their letter which raises several interesting points. We value their contributions to this discussion.

Failure of cerebral autoregulation may be an important step in the cerebral malaria disease process. Ideally our review would have not only included comparisons of autoregulatory function in retinal and cerebral vessels, but also other important subjects such as the nature of the blood-tissue barriers, distribution of endothelial receptors, and vessel ultrastructure in retina and brain. Comparing and contrasting the retina with other areas of the CNS in terms of these and other features could well provide valuable insights, not just into cerebral malaria, but for a whole range of neurovascular diseases. We hope our paper will help to stimulate individual reviews on these topics.

The utility of *Plasmodium falciparum* histidine-rich protein 2 (*pf*HRP2) as a biomarker in severe malaria has recently been reviewed (Manning and Davis, 2013). In African children with severe malaria, high *pf*HRP2 has been associated with anaemia, coma and death (Hendriksen *et al.*, 2012), and distinguishes presence/absence of cerebral sequestration or malarial retinopathy in separate derivation and validation cohorts (Seydel *et al.*, 2012). *pf*HRP2 shows clear promise as an important indicator of *P. falciparum* pathogenesis, and further evaluation as a biomarker of disease severity seems warranted. However, as with any prospective biomarker, evaluation must consider the biological context (Buyse *et al.*, 2010). For example, some strains of *P. falciparum* do not produce *pf*HRP2 (Gamboa *et al.*, 2010). Estimates of total body parasite load may provide useful information at a population level, but subject level variance in important biological parameters leads to improbable values for individual patients (Hendriksen *et al.*, 2012).

Biological context is equally important when considering retinal features as potential markers of cerebral damage. This concern motivated our review. The available evidence suggests that malarial retinopathy, in the context of clinically defined paediatric cerebral malaria (Newton *et al.*, 1998), does indeed reflect similar disease processes in the brain. Although empirical associations between retinopathy and cerebral histopathology necessarily come from limited populations, similarities between retina and brain in terms of anatomy and physiology imply that these associations are also likely to exist more broadly. This biological context suggests inference of several distinct pathological processes from the retina to the brain is plausible, including sequestration, haemorrhage, blood-tissue barrier breakdown, and ischaemia (MacCormick *et al.*, 2014). Consequently, retinal imaging in severe malaria has the potential to provide information on a range of interdependent pathological processes, taking place within the CNS.

In the same way that parasite detection by microscopy depends on an experienced technician, accurate detection of malarial retinopathy depends on the observer and the method. There is a need for a standardized approach to malarial retinopathy. This will facilitate accurate comparisons between studies, and help to define the characteristics and clinical associations of both malarial retinopathy and cerebral malaria more generally. Progressive technological advances in retinal imaging are likely to increase the utility of retinal observations.

Far from precluding the use of other disease markers, retinal imaging represents an opportunity to assess novel biomarkers, both for their clinical use and to investigate relationships between multiple aspects of cerebral pathogenesis. More work is needed to compare retina–brain biological characteristics, describe empirical associations between retina, brain, and prospective biomarkers (including *pf*HRP2), and to standardize imaging methodology in malaria research.
